# Dust in Western Iran: the emergence of new sources in response to shrinking water bodies

**DOI:** 10.1038/s41598-023-42173-3

**Published:** 2023-09-27

**Authors:** Azar Beyranvand, Ghasem Azizi, Omid Alizadeh, Ali Darvishi Boloorani

**Affiliations:** 1 Department of Physical Geography, University of Tehran, Tehran, Iran; 2 Institute of Geophysics, University of Tehran, Tehran, Iran; 3 Department of Remote Sensing and GIS, University of Tehran, Tehran, Iran

**Keywords:** Climate change, Hydrology, Climate sciences, Natural hazards

## Abstract

We detected sources of dust in the Middle East that contribute to dust events in Western Iran in different seasons. By the analysis of the synoptic data, we identified 309 dusty days in Western Iran during the period 2000–2016. A dusty day is diagnosed if under low horizontal visibility (< 1 km), the dust in suspension is reported at least once a day in at least three synoptic stations. We identified dust sources in the Middle East based on the analysis of the MOD04L2 data from MODIS, the backward HYSPLIT trajectory model, and synoptic conditions. The most influential sources affecting Western Iran are located on the shore and northwest of Lake Tharthar, Hour-al-Azim Marsh, the shore of Razzaza, Habbaniyah Lakes, and West Hammar Marsh, which contributed to 110, 79, 59, 56, and 51 dusty days, respectively. The fluctuation of the surface water area largely contributes to the variability of dusty days in Western Iran. Indeed, the peak dust activity in Western Iran was during the period 2008–2012 in response to the substantial shrinkage of the main water bodies in Iraq. The main sources of dust influencing Western Iran are located in northern and eastern Saudi Arabia in spring, Deir ez-Zur in Syria’s Aleppo and Raqqa in summer, and Syria’s Homs and Al-Hasakah in winter and spring. Sources of dust in Western Iraq and in most parts of entire Iraq have, respectively, led to the formation of summer and spring dust events in Western Iran. Decreased precipitation in the Middle East from autumn 2007 to 2012 and the occurrence of severe droughts have also contributed to the shrinkage of lakes and wetlands, as well as the reduced agricultural productivity in the Middle East, all of which contributed to the intensification of dust activity in Western Iran in recent decades.

## Introduction

Dust is one of the main factors threatening human health, especially in arid and semi-arid regions. Some of the adverse effects of dust are chronic obstructive pulmonary disease and an increase in the number of deaths resulting from respiratory diseases^1^. Dust storms also have adverse socioeconomic impacts and might pose a threat to the ecosystem^2^. They may also cause hazards to the environment by for example damaging oil and gas infrastructures^3^, negatively affecting agricultural activity^4^, and livestock farming^5^. Road transportation accidents due to reduced visibility by dust storms have also been reported^6^.

One of the early comprehensive studies about dust storms and their mechanisms was conducted by Hankin^7^. Due to advances in satellite technology and the availability of extensive global data that are generated in an integrated form, it has been possible to better identify sources of dust and understand the mechanisms of dust storms^8^. The work of Prospero et al. was among the first global studies based on satellite data, which provided an initial map of the global dust sources^9^. Tanaka and Chiba used numerical models to estimate the amount of dust emission from different sources across the world^10^. Shao et al. investigated the frequency of dust events in the period 1974–2012 and obtained the spatial distribution of the frequency of global dust^11^. The difference in dust emissions between 2001–2012 and 1980–1996 indicates that most dust sources in the Middle East exhibited an upward trend^12^. The results of Yu et al. also indicate that dust activity in the Middle East increased at a rate of 15% per year for the period from 2000 to 2013^13^.

Sources of dust in the Middle East are noted as the second contributor to global dust load, after North Africa^9^. Most regions of the Middle East are characterized by arid and semi-arid climate, making the region highly vulnerable to the impacts of natural and anthropogenic climate change^14^. Based on the analysis of satellite data for the period 2003–2020, Rezaei et al. find that the highest frequency of dust events in the Middle East occurs at around 45° E over Saudi Arabia, Iraq, and Syria. They conclude that 33, 19, 36, and 12% of dust aerosols in the Middle East are transported toward the west, east, south, and north, respectively^15^.

The identification of dust sources in different regions is vital for better understanding and management of dust storms to partially prevent their hazards to human health and the environment. Numerous studies have been conducted to identify dust sources in the Middle East using different methods^16–23^. They share many common findings, highlighting the significance of dust sources in the Tigris-Euphrates Basin. Among them, Ginoux et al. concluded that most sources of dust in Iraq are natural, whereas those in Syria are mainly the results of human intervention^24^. Nevertheless, only a few studies have attempted to identify the contributing factors to the development of relatively strong dust storms in these sources in recent decades. We argue that the emergence of new sources of dust in the Middle East in response to the shrinkage of water bodies in recent decades may have contributed to more frequent and more severe dust events in this region. We aim to investigate this hypothesis in this study.

At the regional scale, the results of Moridnejad et al. demonstrated that human intervention has indeed contributed to the generation of about 40% of the current sources of dust in Iraq^19^. Walker et al. pointed out that the representation of erodible land surfaces in numerical models should be improved to more accurately simulate the evolution of individual dust plumes over Southwest Asia^25^. Based on the analysis of Spinning Enhanced Visible and Infrared Imager (SEVIRI) data at different temporal resolutions for monitoring dust emission over the Middle East, Hennen et al. concluded that at lower temporal resolutions, the number of dust events is underestimated^26^. Kunkelova et al. identified three new sources of dust in Westernmost Asia, which include the central belt of the Arabian Peninsula, the Southern Levant, and Mesopotamia^27^.

Seasonal variation in the frequency of dust events over the Middle East has also been investigated. For example, Furman and Kutiel investigated the seasonal variability of dust events by studying its abundance in the Middle East for the period 1973–1993. Their results indicate that the highest frequency of dust occurs in summer in Iran, northeastern Iraq, Syria, the Persian Gulf, and Saudi Arabia. Meanwhile, Western Iraq and Syria, Lebanon, northern Saudi Arabia, and southern Egypt experience the highest frequency of dust storms in spring^28^. The results of several studies indicate that the highest frequency of dust storms in Iran is in summer, followed by spring^21,29–32^. Mobark Hassan and Alizadeh also highlight substantial diurnal, seasonal, and interannual variations in the frequency of dust events in southwestern Iran^33^.

The relative importance of dust sources that influence western Iran has not been well addressed in previous studies^17,18,20,23^. In some other studies, seasonal variation in the frequency of dust events in Iran has been investigated^21,29–33^, while seasonal variation in the activity of dust sources that influence Iran is lacking. As such, we have investigated seasonal variation in the activity of dust sources that influence western Iran in recent decades. In this study, by considering a large number of dust storms in western Iran and performing the backward trajectory analysis, we have tried to identify sources of dust that influence western Iran in different seasons. To this end, in addition to the analysis of the true-color satellite images (MODIS-RGB) and MODIS-Merged DT-DB AOD (aerosol optical depth), we have also applied the backward HYSPLIT trajectory model and synoptic analysis. In addition, we have discussed environmental factors that influence the activity of dust sources in the region. For example, we have analyzed hydrological and land cover changes that contributed to changes in dust emission from sources of dust in the Middle East. In particular, we have analyzed the interannual variation in the activity of dust sources in the Middle East and its relation to the interannual variation in hydrological and land cover changes in this region using the LANDSAT images and the Moderate Resolution Imaging Spectroradiometer (MODIS) sensor products.

## Methodology

### Study area

The Middle East is part of the global dust belt^9^, acting as the second largest source of dust in the world after the Sahara in Africa^10^. As a natural hazard, dust events have increased the mortality rate in the region^34^. According to Shao et al., the average dust concentration in the Middle East experienced an increasing trend for the period 1974–2012. An increasing trend has also been identified since the early 1980s but changed into a decreasing trend in the early 1990s. Dust concentrations were reduced to their lowest values between 1993 and 2000, however, the trend has been increasing since then^11^.

The launch of the Güneydoğu Anadolu Projesi (GAP) by Turkey in the 1970s, which aimed to build 22 dams and 19 hydropower plants upstream of the Tigris and Euphrates rivers^35^, has led to a substantial decrease in the downstream water resources in Syria and Iraq since 1990. As a result, agricultural lands in Syria and Iraq decreased from 640,000 hectares to 240,000 hectares^36^. Meanwhile, the Persian Gulf War (1990–1991) caused significant damage to the water supply, irrigation system, and agricultural infrastructure in Iraq^37^, which have been exacerbated by the subsequent international sanctions on Iraq for 12 consecutive years. These have led to a significant reduction in agricultural activity from 2000 to 2011 in Iraq^38^. In addition, recent droughts in the Middle East^39^ have weakened the region's water resources. The expansion of barren lands following the saltation of abandoned agricultural lands, the drying up of lakes, and the semi-arid to the arid climate of the region have created favorable conditions for the intensification of dust storms over the region.

### Identification of sources of dust influencing Western Iran

**Figure 1:**
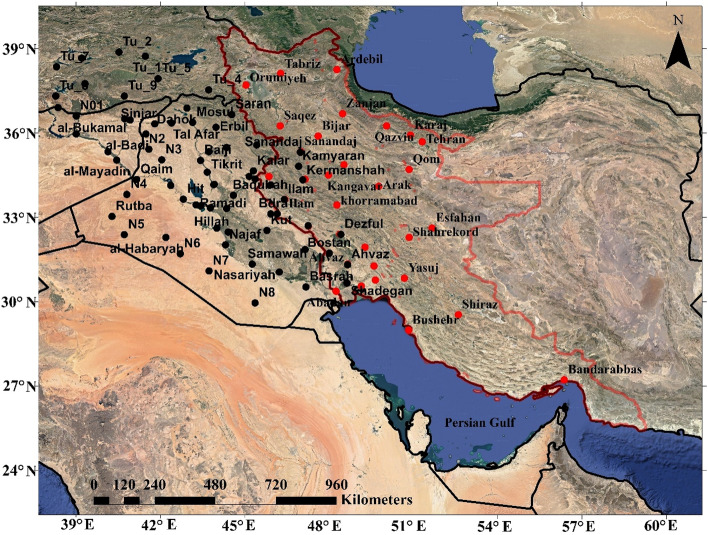
The study area in the Middle East. The synoptic stations whose data were used in this study to identify dust events are marked by filled red circles. Those locations whose precipitation time series were examined are presented by filled black circles, in which points far from cities are named with letters. Precipitation data were taken from the 3B43 algorithm^40^ of the Tropical Rainfall Measuring Mission (TRMM) satellite. Western Iran is shown by red boundaries. We used ArcGIS10.3 to generate the figure.

**Figure 2:**
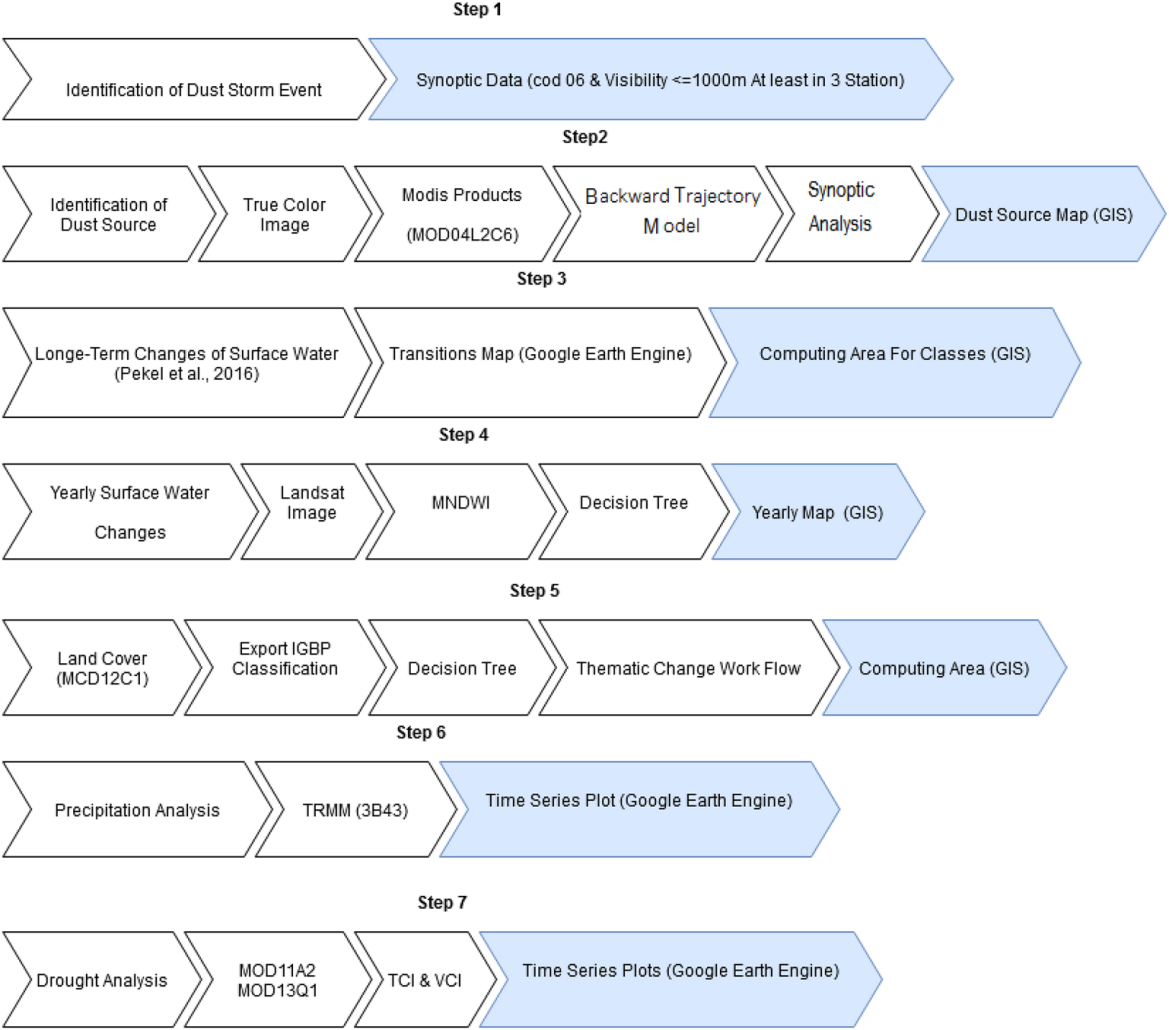
Different conducted steps to identify sources of dust in the Middle East that influence western Iran.

Beyranvand et al. argue that while relatively severe dust storms are dominant in eastern Iran, dust in suspension is the predominant dust phenomenon in western Iran^30^. Accordingly, only dust in suspension phenomenon in western Iran is analyzed in this study. Note that western Iran in this study refers to the western half of Iran, which encompasses northwest, west, and southwest Iran. To identify dust events in western Iran during the period 2000–2016, meteorological data from 33 synoptic stations were obtained from the Meteorological Organization of Iran (see Fig. 1 for the locations of the stations). Similar to Boloorani et al., a particular day is considered a dusty day if at least once a day the horizontal visibility in a station becomes less than or equal to 1 km due to dust in suspension in the present weather phenomenon^18^ (the synoptic code 06 is considered which represents dust in suspension). This condition should be reported at least in three synoptic stations within a distance of 100 km to be considered a dusty day. It should be noted that although according to the definition of the World Meteorological Organization (WMO), a dusty day may include all dust events including dust in suspension, blowing dust, and dust storm, we have only considered the occurrence of dust in suspension with a substantial decrease of horizontal visibility (i.e. less than or equal to 1 km) over a relatively large area (i.e. to be reported at least in three synoptic stations within a distance of 100 km). This is because we intend to only consider those dust events that cover a relatively large region rather than those caused by local sources of dust. Note that in previous studies, horizontal visibility of less than 1 km was also regarded as an important factor to diagnose dust storms^41,42^. Based on this approach, 309 dusty days are diagnosed in western Iran during the study period (Fig. 2—Step 1).

There are three main methods for identifying dust sources in a given region: (1) observing the frequency of dust storms; (2) simulating dust events using numerical models; and (3) the application of remote sensing, visual image interpretation, and digital image analysis. Each of these methods has certain flaws that may cause uncertainty^43^. Despite the existing limitations in ground-based measurements^44^, such as the sparse surface measurement network in remote deserts^43^, data gaps, and LIDAR measurement limitations^45^, large-scale studies have been possible following advances in remote sensing technologies. Indeed, satellite data have been successfully used to trace dust storms and identify their sources^43^. Moreover, satellite data can be used for monitoring the spatial and temporal variability of dust events^46–48^.

In this study, we used the daily MODIS-Merged DT–DB AOD products from the Terra satellite, called MOD04 L2, with a spatial resolution of 10 km, which are based on three algorithms. We used Deep Blue (DB) and Dark Target (DT) algorithms for the land surface (DT for dark land and vegetation and DB for bright and desert surfaces) and the Dark Target algorithm for oceans with a spatial resolution of 10 km. The simple integration of these three algorithms makes it possible to better represent AOD as a map in the transition areas between deserts and regions with relatively dense vegetation such as forests^49^. The monthly mean Normalized Difference Vegetation Index (NDVI) was used to determine the algorithm employed for the surface. When the NDVI is less than 0.2, the DB algorithm was employed, while when the NDVI is greater than 0.3, the DT algorithm was used to retrieve aerosols^49,50^. The Hybrid-Single Particle Lagrangian Integrated Trajectory (HYSPLIT) model^51^ on 1° × 1° GDAS (Global Data Assimilation System) meteorological data was also used to identify sources of dust. To evaluate the MODIS dust products, we tried to apply the AERONET data. However, AERONET stations in the study area are few and there are too many gaps in the data. The SEVIRI images are also not available for the entire study period. However, MODIS is recognized as the best sensor for dust source identification with qualified visual interpretation^52^.

Altogether, 287 backward trajectories by the HYSPLIT model were conducted for 156 dusty days in Western Iran. Then, the mean center and the standard deviational ellipse with one standard deviation were depicted in a geographic information system (GIS). As weather systems enter Iran from the west, the backward trajectory was mainly conducted for synoptic stations located in western Iran, including Ahwaz, Bostan, Dehloran, and Sarpol-e Zahab. The main pathways of dust toward western Iran were determined based on this backward trajectory analysis. However, only strong dust events and the dominant transport pathways were analyzed.

Geopotential height and both zonal and meridional components of wind speed were obtained from the European Centre for Medium-Range Weather Forecasts (ECMWF) reanalysis Interim (ERA-Interim) data with a horizontal resolution of 0.75° × 0.75° and a 6-hourly temporal interval for the period 2000–2016. For the identified 309 dusty days in western Iran during the period 2000–2016, we also analyzed synoptic conditions.

To identify the real sources of dust that influence western Iran, we first identify dusty days in western Iran based on the approach described earlier. Sources of dust in the Middle East are then determined for the identified dusty days in western Iran by diagnosing high-AOD areas in the Middle East using the MOD04 L2 product, examining true-color images (MODIS-RGB), using the backward HYSPLIT trajectory model, and by visual interpretation and synoptic analysis. Figure 2 demonstrates different steps that were conducted to identify sources of dust in the Middle East that influence western Iran. It should be noted, however, that as MODIS products are produced on a daily timescale, they cannot well detect those dust events that evolve on an hourly timescale.

### Dry water bodies

We used Landsat 7 satellite images to investigate water conditions that act as dust sources in the Middle East. We also applied the Google Earth Engine to process the information from these satellite images. The modified normalized difference water index (MNDWI) was used to identify water bodies on the annual timescale (step 4 in Fig. 2). Images that are used to calculate this index in Google Earth Engine are Surface Reflectance Tier 1^53^, for which pre-processing and initial corrections are not required. Then, the decision tree algorithm was used to extract water bodies in different years during the period 2000–2016. Note that the normalized difference water index (NDWI) fails to identify water zones to a satisfactory level (McFeeters, 1996). Therefore, MNDWI (Eq. 1) was introduced by Xu to improve the performance of the NDWI by using the mid-infrared band instead of the near-infrared band. The output of this remote sensing index is between − 1 and + 1, in which values above + 0.5 indicate water bodies^54^.

1 $$\text{MNDWI}=\frac{G-MIR}{G+MIR},$$

where *G* is the green band like band 2 and *MIR* is the mid-infrared band like band 5 in the Thematic Mapper (TM).

Changes in the land surface classes (water/land) in the identified sources of dust in 2015 compared to 1984 are extracted based on the approach of Pekel et al.^55^. They classified the changes in the global surface water into 11 categories, including permanent or seasonal water sources, new permanent or seasonal water sources, dried permanent or seasonal water sources, temporary permanent or seasonal water sources, permanent-to-seasonal water sources, seasonal-to-permanent water sources, and lands with no changes (Fig. 2—step 3).

### Land cover changes

Changes in land cover due to natural or anthropogenic climate change can have significant effects on ecosystems on local, regional, and even global scales^56^. By modifying energy and humidity budgets at the local scale, changes in land cover significantly influence the dynamic and thermodynamic properties of the atmosphere^57^. Therefore, if changes in the surface facilitate soil erosion, such that more soil particles can be entrained, it can create suitable conditions for dust events. In this study, the annual land cover data in source regions in the period 2001–2016 are based on the IGBP classification of the MODIS land cover product (MCD12C1), with a spatial resolution of 500 m. Out of 17 classes of this classification, water bodies, rangelands, savannas, grasslands, agricultural areas, permanent wetlands, and desert areas are identified in the source regions. Using the decision tree algorithm, land surface classes were extracted for each year. Then, changes in land cover in each year were compared against changes in the previous year (Fig. 2—step 5).

The vegetation cover index (VCI) of the MODIS MOD13Q1 product is presented as a time series. This product is generated for 16 days with a spatial resolution of 250 m. This index is calculated using the NDVI and its minimum and maximum values using the following equation^58,59^:

2 $$\text{VCI}=100\times \frac{\text{NDVI}-{NDVI}_{min}}{{NDVI}_{max}-{NDVI}_{min}},$$

where VCI differentiates between NDVI fluctuations, which are associated with short-term meteorological and long-term ecosystem change^58^. The temperature condition index (TCI) of the MODIS MOD11A2 product is also presented as a time series. This product is generated with an 8-day temporal resolution and a spatial resolution of 1 km. The index is calculated using the brightness temperature (BT) and its minimum and maximum values through the following equation^58,59^:

3 $$\text{TCI}=100\times \frac{{BT}_{max}-BT}{{BT}_{max}-{BT}_{min}},$$

where VCI and TCI, respectively, reflect humidity and temperature conditions of the vegetation cover, ranging between 0 and 100%, which denote very poor to optimal conditions, respectively^60^ (Fig. 2—step 7).

The classification of drought conditions based on these indices is presented in Table 1.

**Table 1:** The classification of drought conditions based on the temperature condition index (TCI, %) and the vegetation cover index (VCI, %)^61^.

VCI, TCI values	$<10$	$10\text{ to }19.9$	$20\text{ to }29.9$	$30\text{ to }39.9$	$40\ge$
Drought condition	Extreme	Severe	Moderate	Mild	No Drought

Precipitation data were taken from the 3B43 algorithm of the Tropical Rainfall Measuring Mission (TRMM) satellite and were used to determine those days with precipitation in the study region (see Fig. 1 for the location of those points whose precipitation time series were examined). This algorithm estimates monthly precipitation using a combination of 3-h microwave-3 data and cumulative precipitation data from an infrared sensor^40^ (Fig. 2—step 6). The extraction of precipitation, VCI, and TCI time series in the Google Earth Engine is based on the analysis of the abovementioned images.

## Results

Our analysis indicates that 309 dusty days occurred in western Iran during the period 2000–2016. According to Table 2, the highest number of dusty days in western Iran occurred in 2008, but the lowest was in 2001, 2002, and 2014.

**Table 2:** The number of dusty days in western Iran for the period 2000–2016.

Year	Dusty days	Year	Dusty days
2000	21	2009	39
2001	2	2010	29
2002	2	2011	26
2003	8	2012	31
2004	8	2013	8
2005	28	2014	2
2006	10	2015	13
2007	14	2016	15
2008	52		

### Spatial–temporal analysis of the sources of dust influencing Western Iran

**Figure 3:**
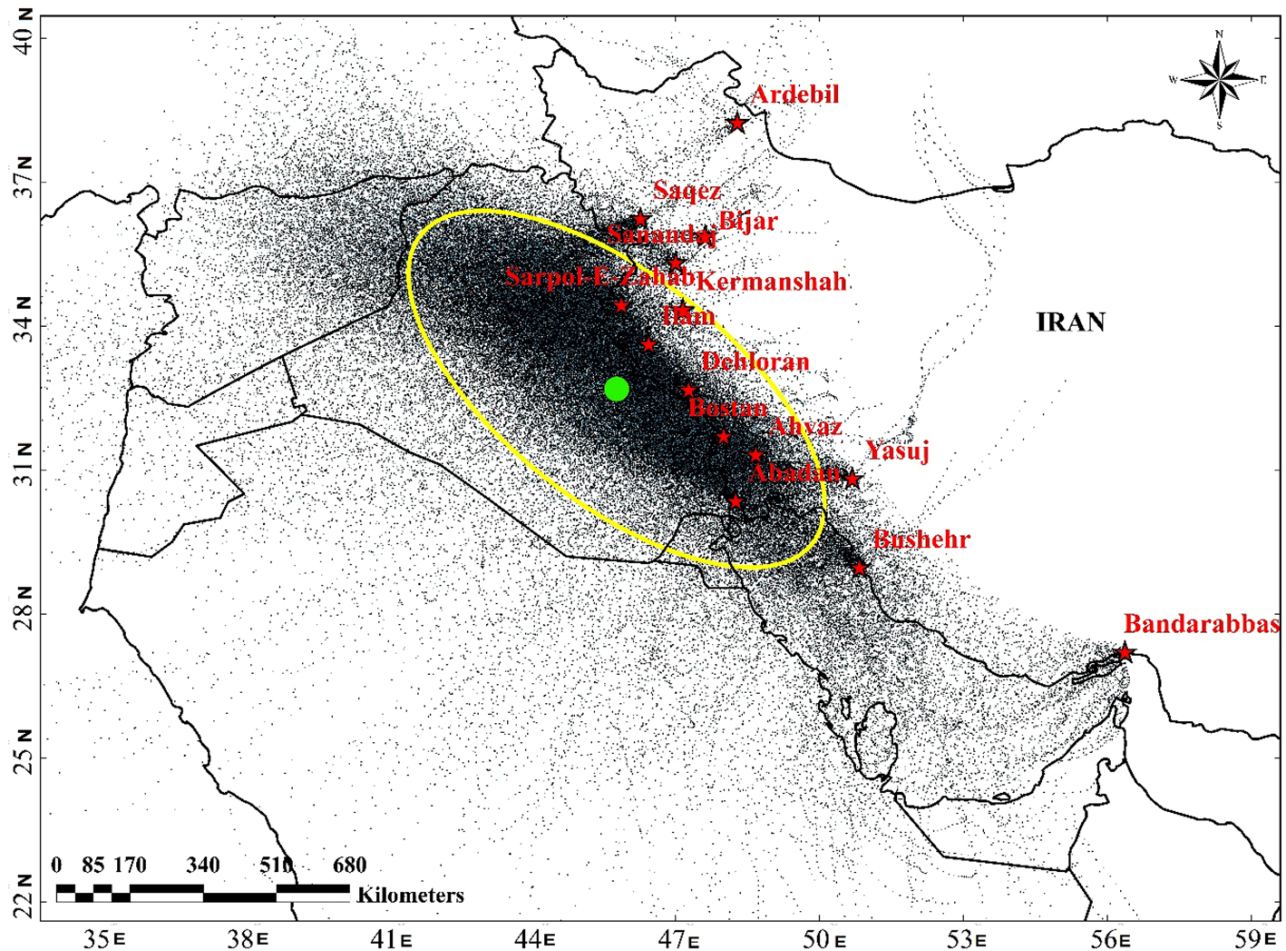
The analysis of the 287 backward trajectories by the HYSPLIT model for 156 dusty days in western Iran that occurred between 2005 and 2016. The mean center (green dot) and the standard deviational ellipse (yellow) with one standard deviation for the backward trajectory analysis are also depicted. Backward trajectories were run from synoptic stations in Iran (red stars). The Global Data Assimilation System (GDAS) data were used for the backward trajectory. We first obtained the routing points from https://www.ready.noaa.gov/hypub-bin/trajtype.pl and then applied ArcGIS 10.3 to generate the figure.

After the identification of dusty days, based on the analysis of the MODIS-Merged DT–DB AOD, true-color images (MODIS-RGB), the backward HYSPLIT trajectory model, and synoptic analysis, sources of dust that influence western Iran are identified. Several dust events are analyzed in this section. The analysis of the 287 backward trajectories for 156 dusty days by the HYSPLIT model is summarized in Fig. 3. The mean center and the standard deviational ellipse with one standard deviation for the backward trajectory analysis are also depicted in Fig. 3. The ellipse is directed northwesterly–southeasterly and covers most parts of Iraq, and the mean center is also located in Iraq. However, we only provide the synoptic analysis and the backward trajectory for some of these dust events that were strong and influenced larger areas in western Iran.

**Figure 4:**
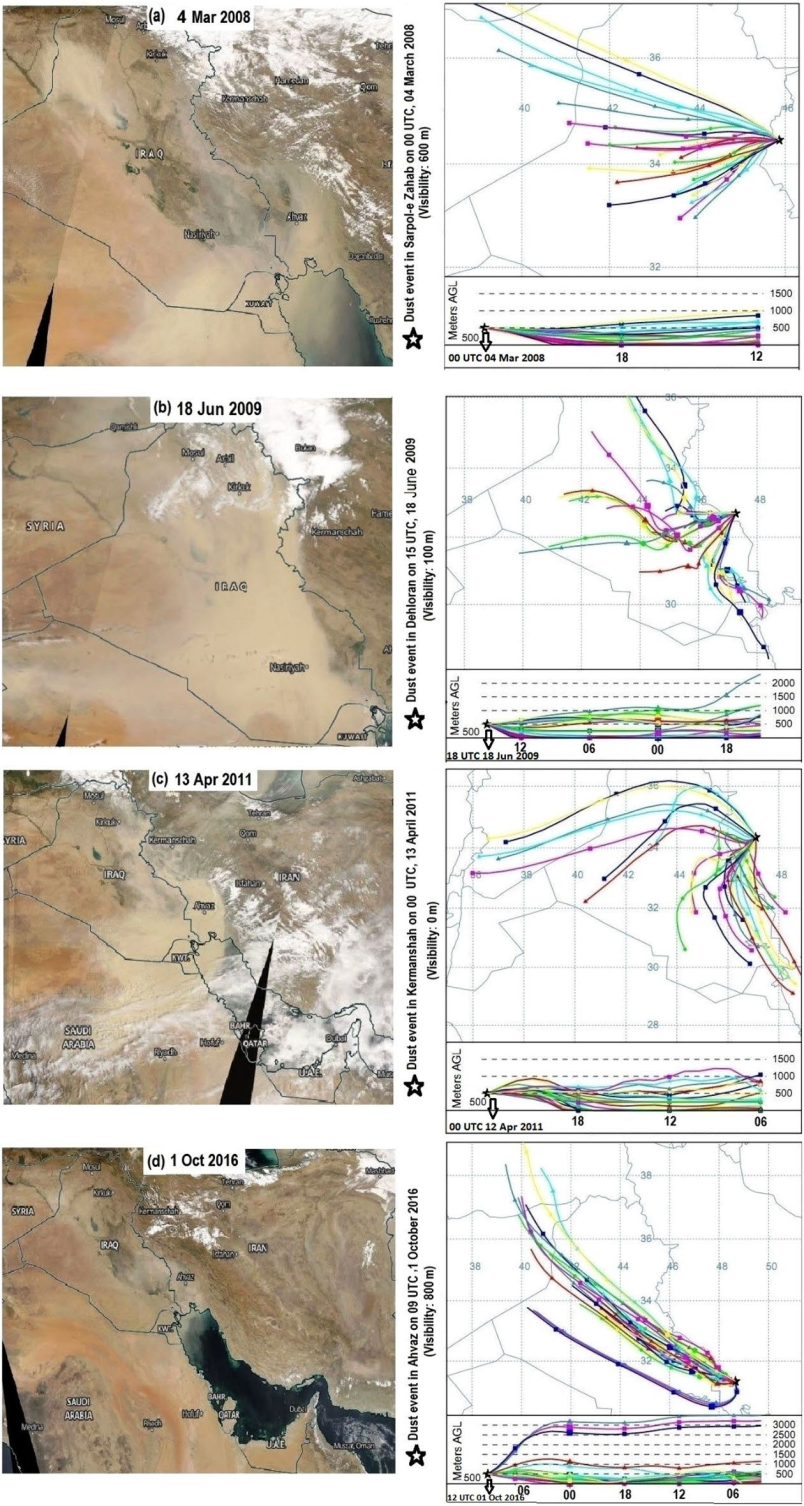
(Left panels) Color images of the MODIS-RGB which show the occurrence of dust events in the Middle East. (Right panels) The backward HYSPLIT trajectory model at some synoptic stations (marked by stars) in western Iran over which horizontal visibility was significantly low. The triangle and square in the right panels are included for every 6 h. AGL denotes above the ground level and the numbers in maps represent the longitude and latitude. We conducted the backward trajectory at 500 m above the ground for 12 h in (**a**), 24 and 18 h in (**b,c**), respectively, and 30 h in (**d**). The duration of the dust event in the top panels was 1 day and dust was reported in 7 stations in Iran. In the upper middle panels, dust was reported in 14 stations and persisted for 3 days, while in the lower middle panels, dust persisted for 2 days and was reported at 20 stations. In the bottom panels, dust was reported in 8 stations and persisted for 2 days. The HYSPLIT trajectories were plotted using the following link: https://www.ready.noaa.gov/hypub-bin/trajtype.pl.

**Figure 5:**
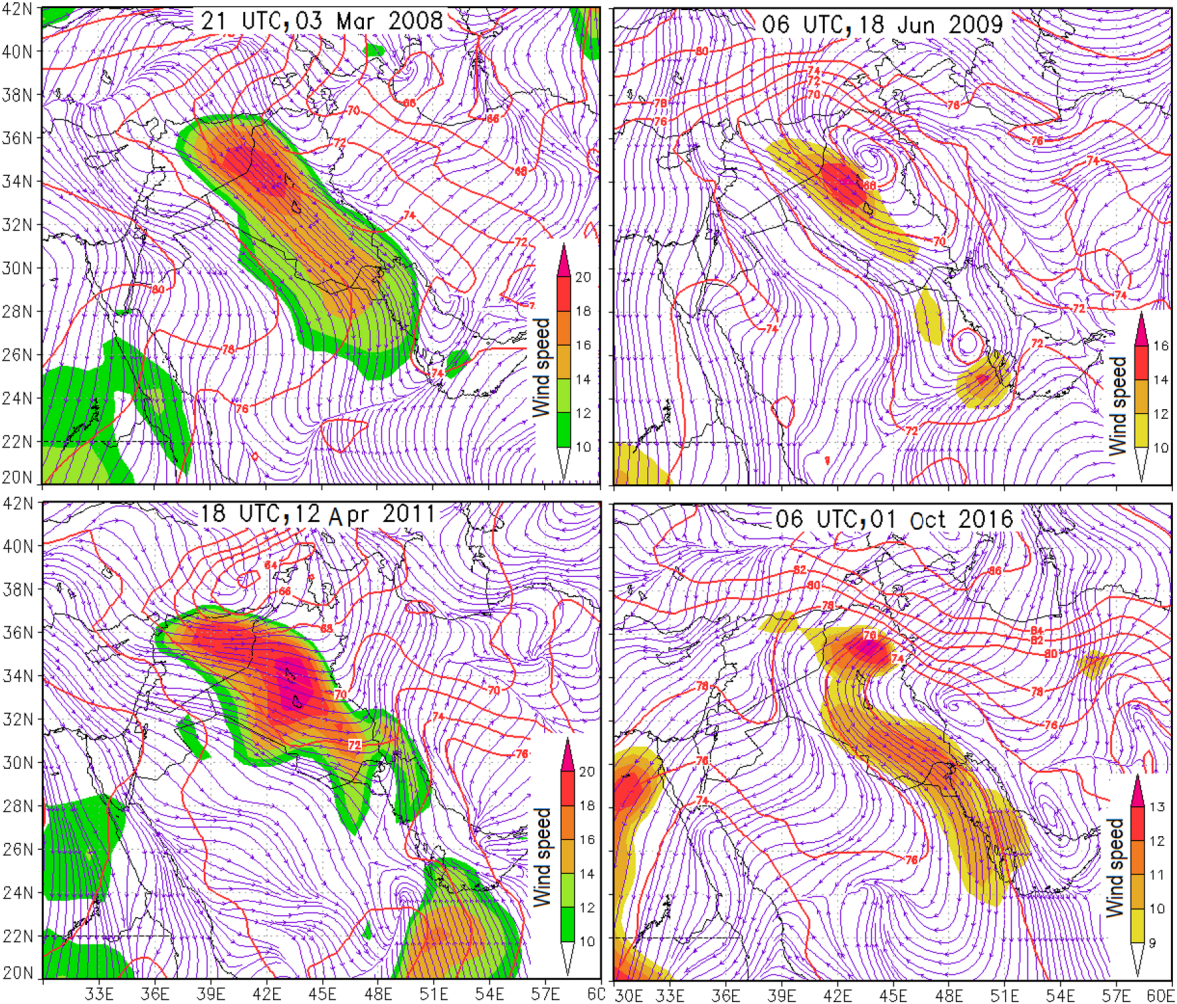
Wind speed (m s^−1^, shaded), geopotential height (red contours with the interval of decameter), and wind streamlines (purple) at 925 hPa. We used open GrADS to generate the figure.

On 4 March 2004, dust storms over Iraq, Kuwait, and Saudi Arabia contributed to the transport of dust toward Iran. According to Fig. 4a, the backward trajectory is analyzed for Sarpol-e Zahab in western Iran where horizontal visibility reached less than 1 km due to dust. Figure 5 also indicates relatively strong wind speeds (about 18 m s^−1^) in the lower troposphere over sources of dust in Iraq and Syria.

The analysis of another dust event on 17–19 June 2009 also indicates the transport of dust toward western Iran from Iraq, Syria, Kuwait, and Saudi Arabia (Fig. 4b). Relatively strong wind speeds in the lower troposphere are also visible over these regions (Fig. 5). The backward trajectory analysis for other dust events in western Iran on 13–14 April 2011 and 1 October 2016 also indicates that dust in western Iran mainly originated from sources of dust in Iraq (Fig. 4c,d).

**Figure 6:**
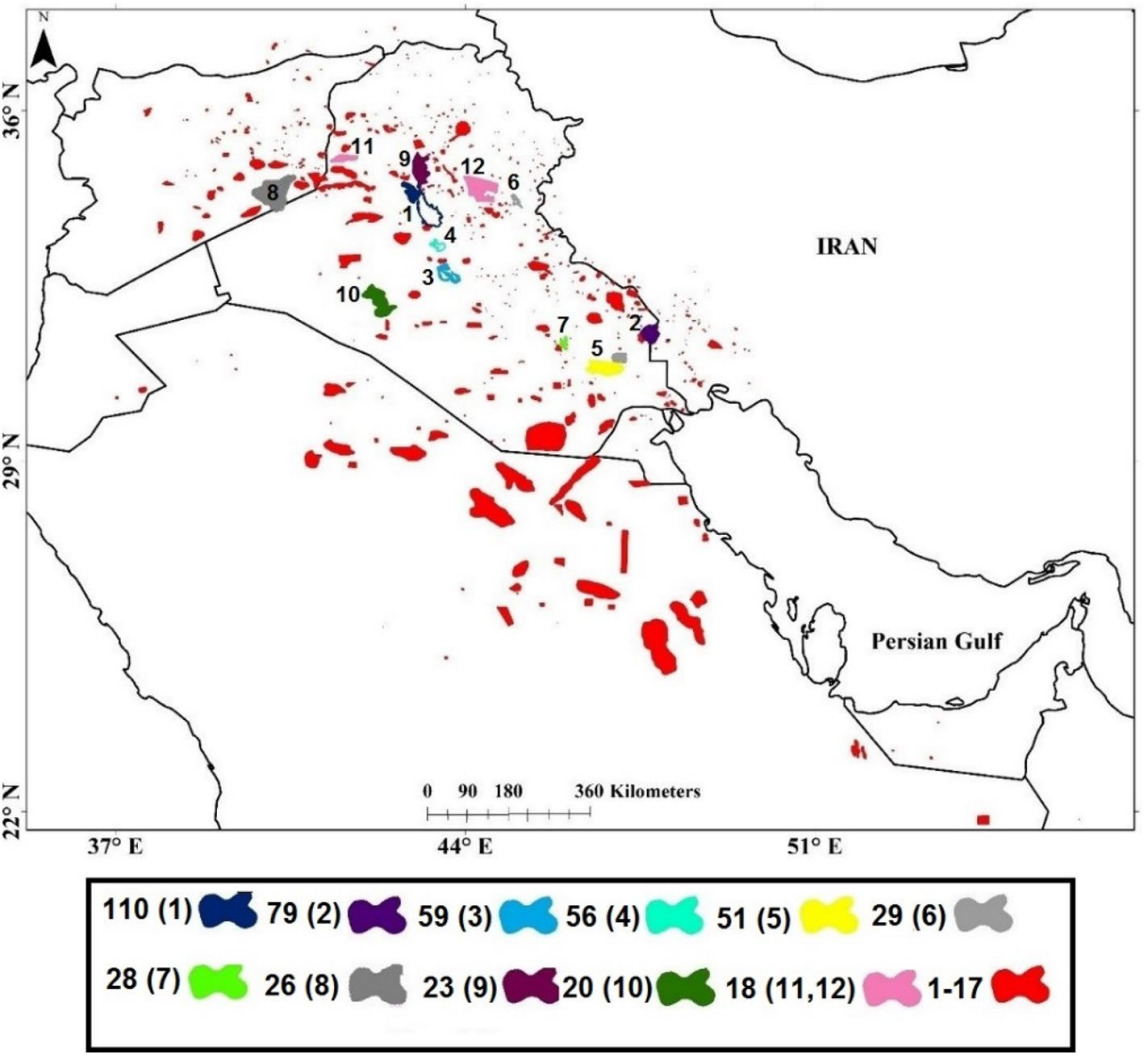
The identified sources of dust in the Middle East that influence western Iran. Colors and associated numbers in the label bar indicate the contribution of each source of dust to the number of dusty days in western Iran during the period 2000–2016. Numbers in parentheses of the label bar which are also shown on the map represent the most important sources of dust that influence western Iran, in which a lower number indicates that the source of dust contributes to a higher number of dusty days in western Iran. We used Google Earth and ArcGIS10.3 to generate the figure.

Figure 6 shows the geographical locations of dust sources in the Middle East that influence western Iran. These sources are mostly located in Iraq, Syria, and the northern half of Saudi Arabia, although a few sources of dust are also identified in Kuwait, Iran, Jordan, the United Arab Emirates (UAE), Oman, and Turkey. Iraq, Syria, and Saudi Arabia, respectively, have been ranked as the most important sources of dust affecting western Iran. Dry bed lakes in central Iraq, Hour al-Azim, the wetlands to the east of Thi-Qar governorate in Iraq (western Hammar marsh), the Syrian Desert, and desert areas in Iraq and northeastern Saudi Arabia have been persistent sources of dust, with a relatively high frequency of dust occurrences during the study period. It should be noted that the identified sources of dust do not have the same contribution to the number of dusty days in western Iran. Sources of dust that contributed to at least 18 dusty days in western Iran are numbered in Fig. 6, in which the lower numbers indicate that the dust source contributes to a higher frequency of dusty days in western Iran.

Our analysis indicates that sources of dust in Oman, the UAE, and southern Saudi Arabia only contributed to dust events in southwestern Iran. The shore and northwestern part of Lake Tharthar, Hour al-Azim Marsh, the shores of Razzaza and Habbaniyah Lakes, and western Hammar Marsh are the most important sources of dust that influence western Iran, which contributed to 110, 79, 59, 56, and 51 dusty days in western Iran, respectively. Overall, according to Fig. 6, sources of dust that influence western Iran in the order of importance are Lake Tharthar (source number 1), Hour al-Azim (source number 2), Lake Razzaza (source number 3), Lake Habbaniyah (source number 4), the West Hammar Marshes (source number 5), Lake Hemrin (source number 6), West of Thi-Qar (source number 7), the Syrian Desert (source number 8), West of Salah al-Din (source number 9), South of Al-Anbar (source number 10), Southwest of Ninawa (source number 11) and East of Salah al-Din (source number 12). The identified sources of dust influencing western Iran in this study are mostly consistent with several previous studies^17,18,20,23^, as shown in Fig. 1 in the Supplementary Material.

The sources of dust that influenced Western Iran were most active in 2008 and 2009. The shore and northwestern part of Lake Tharthar, Hour al-Azim Marsh, the shores of Razzaza and Habbaniyah Lake,s and western Hammar Marsh, respectively, contributed to 30, 26, 25, 19, and 16 dusty days in 2008 and 23, 18, 15, 13, and 14 dusty days in 2009. In some years, however, these sources had little contribution to dusty days in Western Iran. For instance, sources numbered 1, 3, 4, 5, 6, and 7 (Fig. 6) did not play a significant role in dusty days in Western Iran in 2013 (not shown).

The most important sources of dust in Syria only influenced Western Iran in some particle years such as in 2008 and 2009. Sources of dust in Jordan also only contributed to dusty days in Western Iran in 2015 (not shown).

### Seasonal analysis of sources of dust influencing Western Iran

**Figure 7:**
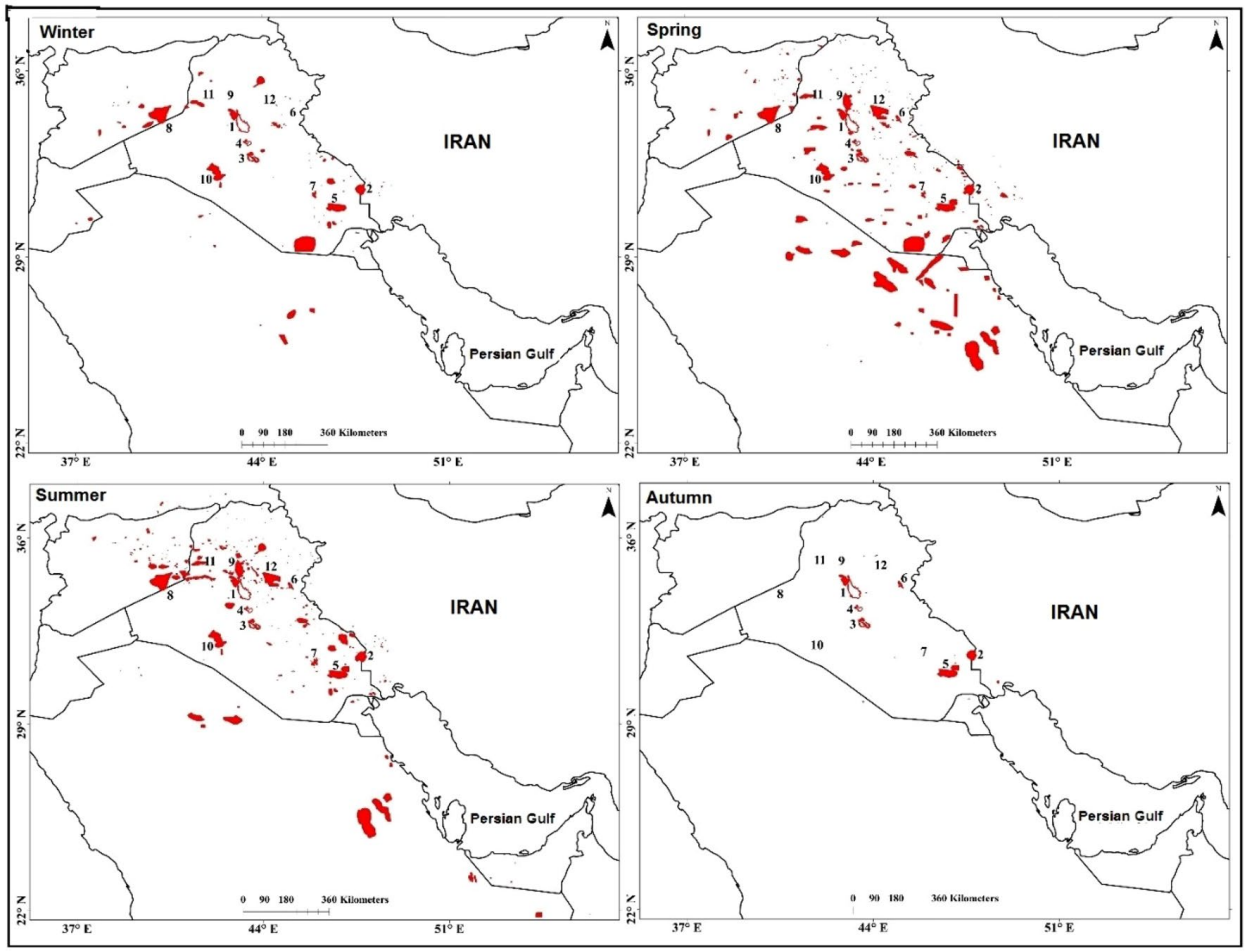
Sources of dust in the Middle East influencing western Iran in different seasons. Numbers in the panels represent the most important sources of dust that influence western Iran. A lower number indicates that the source of dust contributes to a higher number of dusty days in western Iran. We used ArcGIS10.3 to generate the figure.

Figure 7 shows sources of dust in the Middle East that contribute to dust events in western Iran in different seasons. Here, the seasons are defined as and winter (December, January, and February), spring (March, April, and May), summer (June, July, and August), autumn (September, October, and November). According to Fig. 7, some of the sources of dust are active in all seasons, while some of them are only active during two or three seasons. Our analysis indicates that sources of dust in the Middle East contributed to a higher number of dusty days in western Iran in summer, followed in decreasing order by spring, winter, and autumn.

Due to the shrinkage of lakes in some years during the study period, the shores of the Tharthar, Razzaza, and Habbaniyah lakes and Hour al-Azim and western Hammar marshes have become dust sources throughout the year. In some years, especially after 2007, these sources have been active even during winter. The highest activity of dust sources numbered 1 to 5 (Fig. 6) was in the summer of 2008. The source of dust on the shore and northwestern part of Lake Tharthar (numbered 1 in Fig. 6) was also highly active in the summer of 2009, 2010, and 2011. The shore of Lake Hemrin has been mostly active in spring, summer, and autumn. Active sources of dust in northern Saudi Arabia have the most contribution to the springtime dusty days in southwestern Iran, as previously also noted by Furman and Kutiel and Notaro et al.^28,62^. Meanwhile, the highest winter dust activity in Saudi Arabia occurs in its southern part^63^, which generally does not affect Iran. The highest activity occurred in Deir ez-Zur, Aleppo, and Ar Raqqah in summer and Homs and Al-Hasakah in winter and spring. The activity of summer sources in Iraq was mostly concentrated in the Salah al-Din, Naynawa, and northern Al-Anbar governorates, whereas the springtime sources are also active in other parts of Iraq. Dust events in Iraq in summer are generally driven by the so-called Shamal wind^21,64^, which blows in the floodplains of the Tigris and Euphrates rivers, causing the emission of dust particles and their transport toward Iran. Kutiel and Furman examined dust storms in the Middle East in the period 1973–1993. According to their findings, dust storms are most active in northeastern Iraq during summer and in western Iraq during spring, which is not consistent with our results probably because our analysis has been conducted during a different period^28^.

**Figure 8:**
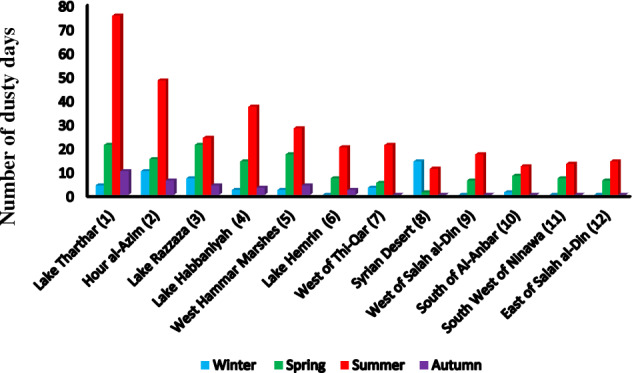
The number of dusty days in western Iran that originated from different major sources of dust in the Middle East in different seasons.

Sources of dust in the Middle East that influence western Iran show large seasonal variability, with the most activity during summer, followed in decreasing order by spring, autumn, and winter when averaged over all dust sources (Fig. 8). Specifically, note large differences between the summertime dust activity in Lake Tharthar and Hour al-Azim compared to the other seasons. The contributions of Tharthar, Razzaza, and Habbaniyah lakes to dusty days in western Iran are, respectively, 68.2%, 40.7%, and 66.1% in summer, 19.1%, 35.6%, and 25% in spring, 9.1%, 4%, and 5.3% in autumn and 3.6%, 7%, and 3.6% in winter. The contribution of western Hammar Marsh to dusty days in western Iran is 54.9% in summer, 33.4% in spring, 7.8% in autumn, and 3.9% in winter, while that of Hour al-Azim Marsh is 60.8% in summer, 19% in spring, 7.5% in autumn, and 12.7% in winter. In winter, Hour al-Azim most significantly contributes to dusty days in western Iran compared to the other sources in the same season, while in autumn Lake Tharthar most significantly contributes to dusty days in western Iran.

### Precipitation and drought in sources of dust

The number of dusty days in western Iran is relatively high in 2000, 2005, 2008–2012, 2015, and 2016 compared to the other years during the period 2000–2016. Our analysis indicates that precipitation decreases in large parts of the Middle East from the autumn of 2007 to the autumn of 2008 and also in 2009 (Figs. 2, 3 in the Supplementary Material). Reduced precipitation was also observed in Iraq (between 2010 and 2012), southern Iraq (2015), and western and southwestern Iran (2012 and 2015). Thus, the decreased precipitation is one of the main contributing factors to a decrease in the number of dusty days in the abovementioned years because reduced precipitation leads to reduced soil moisture that contributes to higher emission of dust in potential sources of dust^65^. A significant negative relationship between precipitation and the frequency of dust was also noted in other parts of the world^66,67^.

**Figure 9:**
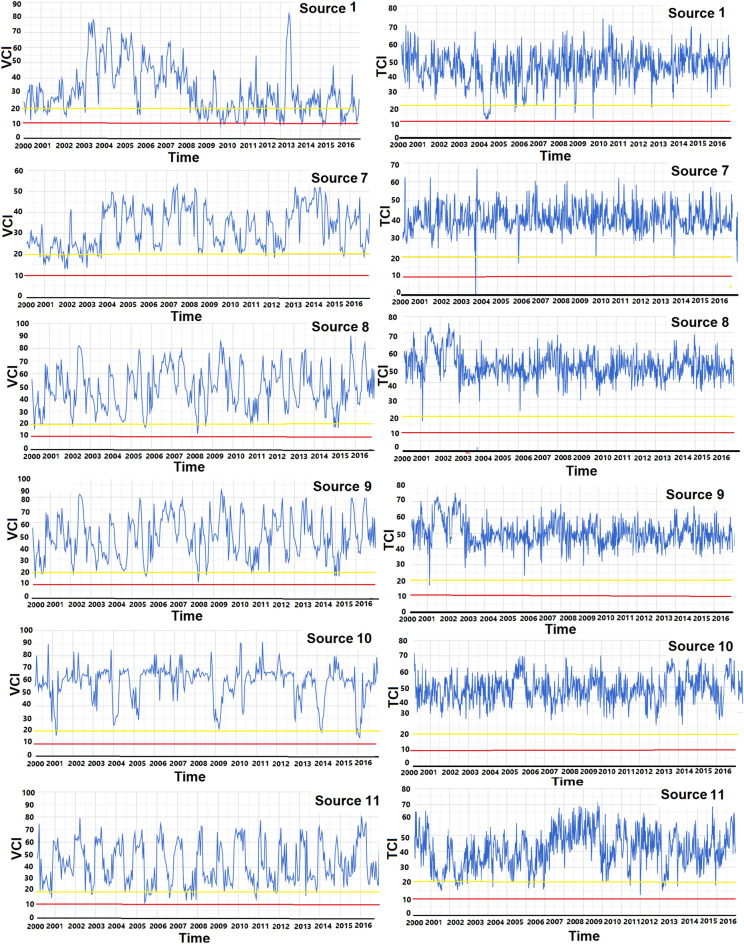
Time series of drought conditions in the main sources of dust in the Middle East based on the analysis of the temperature condition index (TCI, %) and the vegetation cover index (VCI, %). The red lines indicate the occurrence of extreme drought and the yellow lines indicate the occurrence of severe drought.

Other factors contributing to the rise of dusty days in western Iran during particular years are the occurrence of two large-scale droughts in the Middle East^39^ in 1999–2001 and 2007–2008, along with local extreme droughts in different sources and months in 2005, 2008–2012 and 2015 which can be identified based on the analysis of TCI and VCI. Extreme droughts from 2008 to 2012 were more significant in the northwest of Lake Tharthar (source number 1) and the north of Lake Tharthar (source number 9) compared to the other sources of dust. Other examples are extreme droughts in eastern Syria (source number 8) in 2008 and western Naynawa (source number 11) in 2005 and 2010, severe droughts in source number 7 in 2009–2012, an extreme drought in 2015 and 2016, and a severe drought in 2009 in source number 10 (Fig. 9). Indeed, the results of Darvishi Boloorani et al. (2021) indicate that a 34% increase in the frequency of dust events in the Tigris-Euphrates Basin from 2008 to 2012 coincides with a period of drought in the region^68^.

### Dry water bodies that act as sources of dust in the Middle East

Over 70% of the permanent surface water reduction in the globe is concentrated in five countries in Central Asia and the Middle East (Kazakhstan, Uzbekistan, Iran, Afghanistan, and Iraq), with the highest reduction in Kazakhstan and Uzbekistan from 1994 to 2009^55^. According to Peckel et al., 2016, decreases in the permanent surface water in Iran, Afghanistan, and Iraq were respectively by 56, 54, and 34% in 2015 compared to 1984. This dry condition partly contributed to an increase in the activity of dust sources in the Middle East. Our analysis indicates that about 1133.5 km^2^ of regions with permanent surface water in 1984 completely dried up in 2015, which can act as sources of dust, while about 2154.2 km^2^ dried up in some seasons in 2015 compared to 1984. In addition, about 1102.8 km^2^ of the seasonal surface water area in 1984 completely dried up in 2015 (Table 3). Note that according to Table 3, in addition to the lost surface water area, a new water area is created in the year 2015 compared to that in 1984, although the lost area is much larger than the new area.

**Table 3:** Changes in the surface water area of the dried water bodies that act as sources of dust in the Middle East. The changes are computed for the surface water area in 2015 minus that in 1984 (*Source* EC JRC/Google).

Class name	Changes in surface water area (km^2^)
Permanent surface water converted to seasonal water	406
Newly created permanent surface water	138.4
Surface water dried up permanently	1133.5
Surface water that exists for some periods throughout the year	240
Surface water that exists for some periods during some seasons	2154.2
Dried up seasonal surface water	1102.8
Newly created seasonal surface water	1040
Seasonal surface water converted to permanent	12.8

**Figure 10:**
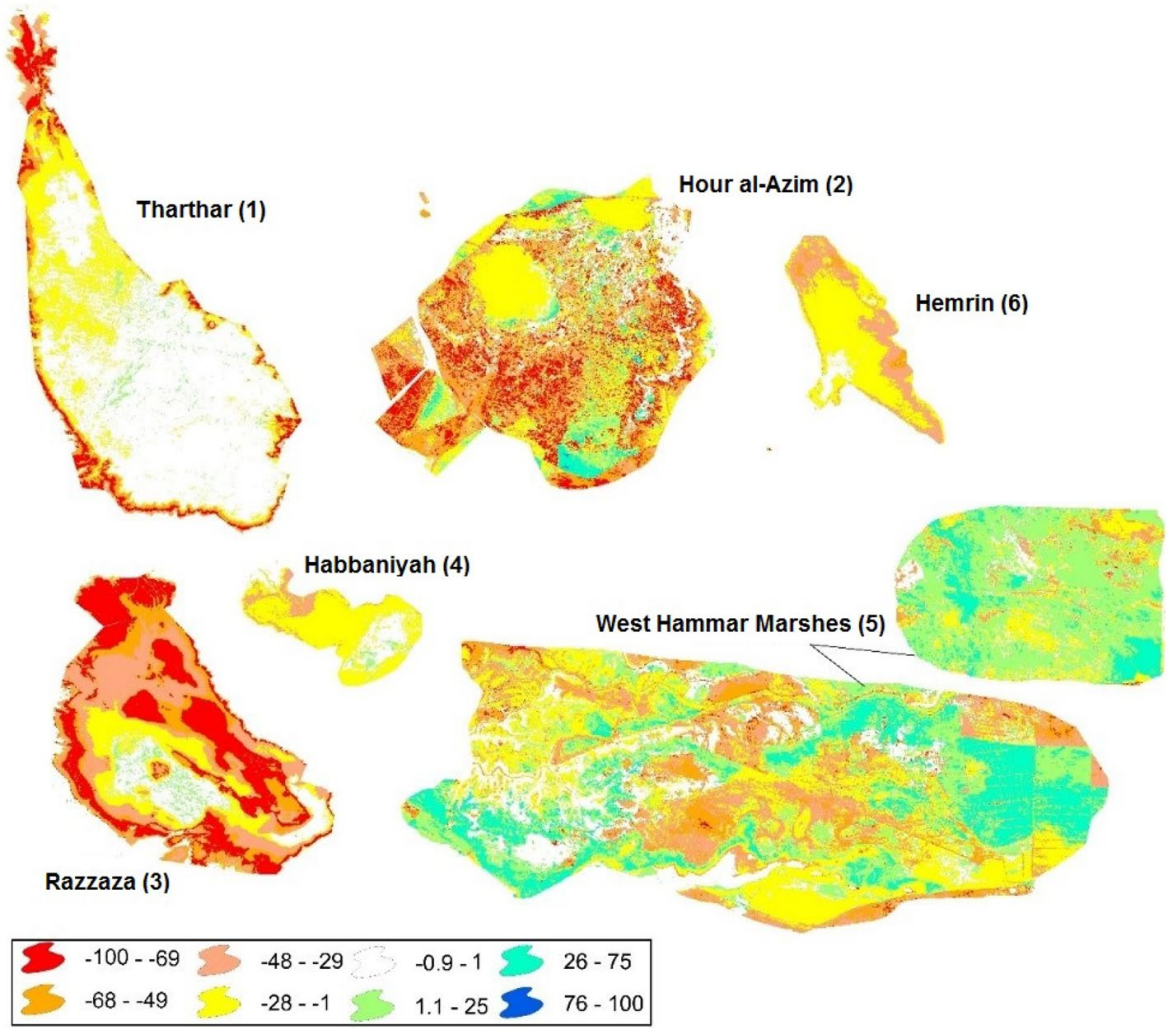
Changes in the surface water (%) of the most important dried water bodies that act as sources of dust in the Middle East that influence western Iran. The changes are computed for the period 2000–2015 minus those in the period 1984–1999. Note that the negative and positive values indicate decreases and increases in the surface water areas in the period 2000–2015 compared to the period 1984–1999 (*Source* EC JRC/Google). We obtained the information layers from Google Earth and used the final processing steps in ENVI 5.0, and generated the figure using ArcGIS 10.3.

Figure 10 shows changes in the surface water of the most important dry water bodies that act as sources of dust in the Middle East that contribute to dust events in western Iran. The changes are computed for the period 2000–2015 minus those in the period 1984–1999. The surface water area of Lake Hemrin decreased by 48% in some places. Lake Tharthar has experienced a 68% decrease across its margins, but reaches up to 100% in some places, although a lower decrease of up to 28% is obtained in the northern half of the lake. Lake Habbaniyah also experienced a decrease in the surface water area in most places. The highest decrease among the main important sources of dust belongs to Lake Razzaza with decreases of around 50%. Both decreases and increases in the surface water can be seen in different parts of both the West Hammar Marsh and Hour-al-Azim Marsh, although decreases are higher.

**Figure 11:**
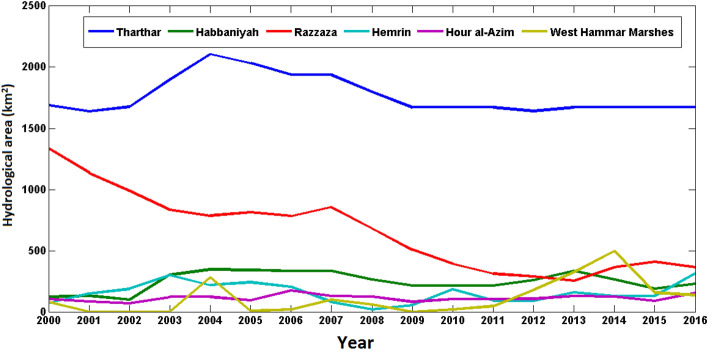
The interannual variability of the surface water area (km^2^) in the dried water bodies that act as sources of dust in the Middle East for the period 2000–2016.

Figure 11 shows the interannual variability of the surface water area in dried water bodies that act as sources of dust in the Middle East for the period 2000–2016. A decrease in the surface water area in Lake Razzaza from 2009 to 2012 is remarkable. Lake Habbaniyah and Lake Tharthar reached their lowest surface water area in 2011, while the lowest surface water area in Lake Hemrin occurred in 2009, and that of Hour al-Azim occurred in 2010 and 2011. A relatively high frequency of dusty days in western Iran from 2008 to 2012^30^ can be partly caused by the discussed decreases in the surface water levels in these dried water bodies that act as sources of dust. An increase in dust activity due to the drying up of lakes has also been reported in some other regions. For example, Indoitu et al. show that severe drying of the Aral Sea contributed to the formation of new sources of dust^69^, which is also highlighted in Nobakht et al.^70^. Rashki et al. investigated the effect of the drying of Lake Hamun on dust events in southeastern Iran and found that the frequency and severity of dust storms significantly increased in the region following a dry period at the end of 1999^71^.

**Figure 12:**
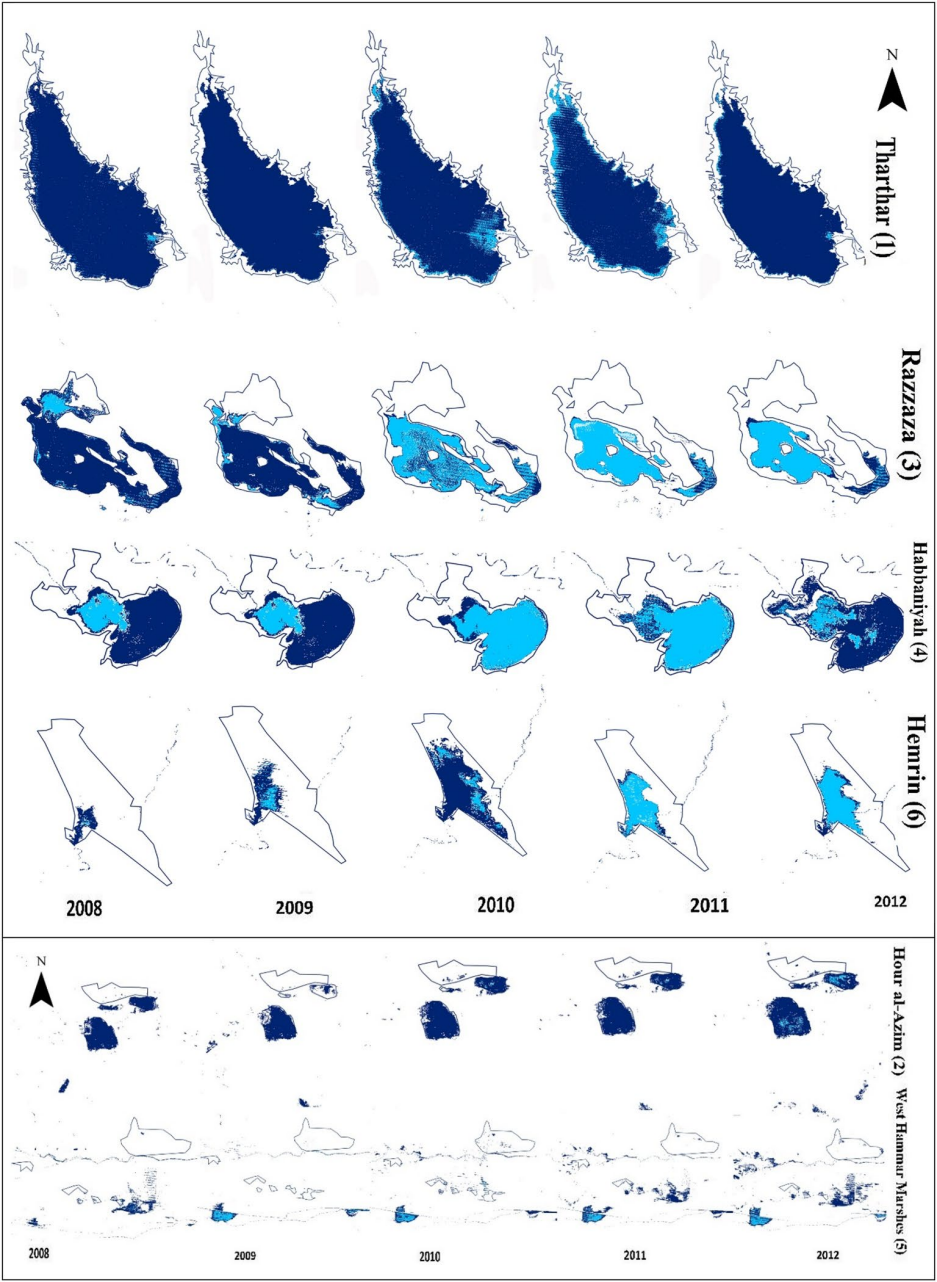
The surface water area (dark blue) of 6 dried water bodies that act as sources of dust in the Middle East for the years from 2008 to 2012. The boundaries of the sources are for the wettest year for each source during the period 2000–2016 during which the surface water area reached the maximum value. These values were obtained based on the modified normalized difference water index (MNDWI), which was extracted from Landsat images. The light blue color shows the fluctuation of the surface water area in different seasons during each year. We calculated the MNDWI in Google Earth then used the final processing steps in ENVI 5.0, and generated the figure using ArcGIS 10.3.

Figure 12 shows the surface water area of 6 dried water bodies that act as sources of dust in the Middle East for the years from 2008 to 2012. Note that the eastern part of Lake Hemrin, the northwestern part of Lake Habbaniyah, the northeastern part of Lake Razzaza, the Shore of Lake Tharthar especially in its northwestern part, the West Hammar Marshes, and the northern part of Hour al-Azim have dried up in these years.

### Impact of land cover change on the activity of dust sources in the Middle East

**Figure 13:**
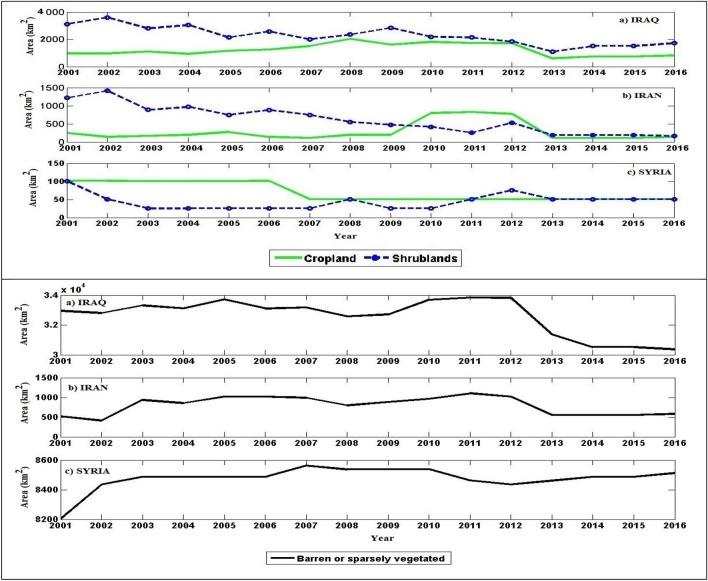
The interannual variation of the area of some land cover classes in the identified sources of dust in Iraq, western Iran, and Syria for the period 2001–2016. The identified sources of dust in these three countries are shown in Fig. 6.

Figure 13 shows the interannual variation of the area of some land cover classes in the identified sources of dust in Iraq, western Iran, and Syria for the period 2001–2016. Barren or sparsely vegetated surfaces reached their greatest area in 2005, 2010, 2011, and 2012 in Iraq, 2007 to 2010 in Syria, and 2011 and 2012 in Iran. A decrease in the area of croplands occurred in Syria from 2006 to 2016, while the area of croplands in the sources of dust in Iraq increased for the period 2008–2012, which was partly due to the drying up of wetlands and their usage as agricultural lands.

Table 4 shows changes in the area of different land cover classes in different sources of dust in the Middle East for successive years during 2000–2016. Between 2010 and 2012, 726.1 km^2^ of the source of dust lost its shrub cover and turned into barren lands. From 2009 to 2010, 248.2 km^2^ of permanent wetlands became croplands. Over 80 km^2^ of croplands and grasslands were also converted into shrublands from 2010 to 2012.

**Table 4:** Changes in the area of different land cover classes in different sources of dust in the Middle East for successive years during 2000–2016. The locations of the sources of dust are shown in Fig. 6.

Class name	Changes in the land cover area (km^2^)														
	2001–2002	2002–2003	2003–2004	2004–2005	2005–2006	2006–2007	2007–2008	2008–2009	2009–2010	2010–2011	2011–2012	2012–2013	2013–2014	2014–2015	2015–2016
Shrubland to barren or sparsely vegetated land	50.2	465		263.4	110.6	282.7		111.34	311.1	138.7	276.3	133.1			
Shrubland to grassland					25.1										
Shrubland to cropland					27.6	82.9	27.6								
Shrubland to permanent wetland						110.4						303.8			
Permanent wetland to barren or sparsely vegetated land					27.6										
Permanent wetland to cropland									248.3						
Cropland to shrubland						25.1		414.53		55.1					
Cropland to grassland										83					
Cropland to permanent wetland												27.6			
Grassland to barren or sparsely vegetated land												25.8			
Grasslands to shrubland	83	25							25.1						
Savannas to permanent wetland												55.1			
Barren or sparsely vegetated to shrubland			179.7		133.4	82.9	238.89	26.9		102.8	27.9		104.5		
Barren or sparsely vegetated to grassland					27.7						154.3				
Barren or sparsely vegetated to croplands					193.7										

## Discussion

### Identification of dust sources

The identification of dust sources is essential in regions that are largely affected by dust events. As most regions of Iran are characterized by semi-arid to arid climate^14^, there are potential sources of dust with adverse socioeconomic impacts. The identification of these sources requires new investigations. However, there are some limitations in this regard, including the lack of enough synoptic and AERONET stations and the inherent uncertainty of satellite^72^.

In this study, we have identified the potential sources of dust that contribute to dust events in western Iran. To this end, we have used meteorological data from synoptic stations and satellite data and conducted backward trajectory analysis by the HYSPLIT model. We have also analyzed seasonal and interannual variations in the activity of the sources of dust in the Middle East, which has not been well addressed in previous studies. To understand a relatively higher activity of the sources of dust in particular years, hydrological and land cover changes have also been analyzed. The identified sources of dust in this study are similar to those of Zoljoodi et al.^17^, Darvishi et al.^18^, Cao et al.^20^, and Darvishi et al.^23^, although different approaches were used in these studies.

### Surface water fluctuation

Drying up a significant percentage of surface water in the Middle East in recent years is a key factor in exacerbating the occurrence of dust events in the Middle East. In particular, our analysis indicates that the fluctuation of the surface water area in Tharthar, Razzaza, Habbaniyah, and Hemrin lakes largely contributed to the fluctuation in the number of dusty days in western Iran, with the peak dust activity during the period 2008–2012 when the surface water of the main dried water bodies that act as sources of dust substantially decreased. It should be noted that in addition to precipitation, water inflow/outflow management by humans also largely affects the fluctuation of the surface water in these lakes. Considering the presence of coastal sediments, the surface water decrease of these lakes largely contributes to an increase in dust activity over the region. Meanwhile, given the dependence of agriculture on the water resources of these lakes in many parts of the region, a decline in the surface water of these lakes may decrease agricultural activities, which may lead to agricultural dust emissions. Our results are consistent with Darvishi Boloorani et al. who explored changes in the water areas of the Tigris-Euphrates Basin^68^.

### Changes in wetlands

Located on the Iran-Iraq border in Khuzestan Province, Hour al-Azim (source number 2 in Fig. 6) receives its water mainly from the Tigris River in Iraq and the Karkheh River in Iran. The operation of several dams constructed on the Tigris River in Turkey and Iraq and on the Karkheh River in Iran in the last decade of the twentieth century has substantially reduced the surface water of this wetland. Moreover, several droughts and reduced precipitation in the region in recent decades have contributed to a decrease in vegetation cover and agricultural activities in parts of this wetland, causing a substantial rise in dust activity.

The West Hammar Marshes (source number 5 in Fig. 6) is part of the Hammar Marsh, which is located in southern Iraq in the Thi-Qar Governorate, which receives its water mainly from the Euphrates River. Recent studies show that the condition of the western Hammar Marsh is worse than other Iraqi and Mesopotamian marshes as it is fed by the Euphrates River which has been substantially shrunk following the construction of dams in recent years^73^. The hydrological conditions of Mesopotamian marshes are closely related to the hydrological conditions upstream of the Tigris and the Euphrates. In the 1990s, the Iraqi government began draining wetlands to control water shortages and to manage drinking and irrigation sectors. Since 2003, following the collapse of the Iraqi government, these wetlands have been fed again^74^. According to the Iraqi Ministry of Water Resources, about 43% of the wetlands were refilled in 2008 and 57% of them are still dry for agricultural or oil extraction activities.

### Land cover changes in sources of dust

The occurrence of extreme droughts from 2008 to 2012 was more significant in the northwest of Lake Tharthar (source number 1 in Fig. 6) and the north of Lake Tharthar (source number 9 in Fig. 6) compared to other sources. Some examples of droughts are an extreme drought in eastern Syria (source number 8 in Fig. 6) in 2008 and western Naynawa (source number 11 in Fig. 6) in 2005 and 2010, a severe drought in source number 7 in Fig. 6 from 2009 to 2012, an extreme drought in 2015 and 2016, and a severe drought in source number 10 in 2009. Considering that most desert areas have poor vegetation, interannual changes in vegetation are determined by droughts^75^. The impact of droughts is particularly remarkable in the Middle East because this region is mostly characterized by barren or sparsely vegetated surfaces. Between 2010 and 2012, a large area of sources of dust in the Middle East lost their shrub cover and became barren lands, or a large fraction of these surfaces with the cropland and grassland were converted into shrublands. The high susceptibility of areas with barren or sparse vegetation cover to short-term droughts^76^ has caused the dominance of a harsh climate in these areas, creating favorable conditions for the intensification of dust events and an increase in their frequency. Meanwhile, the effect of human activities on regional dust sources aggravates the negative effect of droughts, for example by land cover/land use changes^77^. Changes in land cover, especially the conversion of wetlands to other land cover classes, in the region are explored by Bakhtiari et al.^78^. Our results also indicate that changes from wetlands to agriculture or bare surfaces were remarkable from 2010 to 2012. Moridnejad et al. also found that nearly 40% of dust centers in Iraq and Syria have been created due to anthropogenic desertification processes^19^.

## Conclusions

Using 3-hourly meteorological data of 33 synoptic stations in western Iran, 309 dusty days are identified during the period 2000–2016, with the highest number of dusty days in 2008. The MOD04L2 data from the MODIS sensor were analyzed through visual interpretation and 536 dust sources in the Middle East were identified.

The findings indicate that the shore of Lake Tharthar, Hour al-Azim Marsh, Razazeh Lake, Lake Habbaniyah, and the West Hammar Marshes are the sources of dust in the Middle East that influence western Iran. In addition, based on the analysis of 287 backward trajectories for 156 dusty days by the HYSPLIT model, we find that the main sources of dust that influence western Iran are located in Iraq.

Our analysis indicates that a greater number of dust sources were active in Iraq in 2008 and 2009. The shore and northwestern part of Lake Tharthar, Hour al-Azim Marsh, the shores of Razzaza and Habbaniyah Lakes and western Hammar Marsh, respectively, contributed to 30, 26, 25, 19, and 16 dusty days in western Iran in 2008 and 23, 18, 15, 13, and 14 dusty days in 2009.

The highest frequency of dust days in western Iran is in summer, followed by decreasing order by spring, autumn, and winter. Indeed, more than 50% of the contribution of dried water bodies that act as sources of dust on the shores of Lake Tharthar, Habbaniyah, the West Hammar Marshes, and Hour al-Azim were observed in summer. However, the highest effect of sources of dust in Saudi Arabia on the occurrence of dust storms in southwestern Iran was observed in spring. In Syria, the highest dust activity occurred in Deir ez-Zur, Aleppo, and Ar Raqqah in summer, but in Homs and Al-Hasakah in both winter and spring. In summer, the main sources of dust in Iraq were the Salah al-Din, Naynawa, and northern Al-Anbar governorates, whereas some other sources of dust were also active in other parts of Iraq. The contribution of Tharthar, Razzaza, and Habbaniyah lakes on dusty days in western Iran in summer compared to the other seasons is 68.2%, 40.7%, and 66.1%, respectively.

The analysis of the time series of precipitation indicates that precipitation decreases in large parts of the Middle East from the autumn of 2007 to the autumn of 2008 and in the whole of 2009. Precipitation also decreased in Iraq between 2010 and 2012, southern Iraq in 2015, and western and southwestern Iran in 2012 and 2015.

Based on the analysis of TCI and VCI, droughts were more significant in the northwest of Lake Tharthar (source number 1) and the north of Lake Tharthar (source number 9) compared to the other sources of dust from 2008 to 2012. Other examples include an extreme drought in eastern Syria (source number 8) in 2008 and western Naynawa (source number 11) in 2005 and 2010, severe droughts from 2009 to 2012 and also an extreme drought in 2015 and 2016 in source number 7, and a severe drought in source number 10 in 2009.

In addition, decreases in the surface water of lakes in Iraq have led to the emergence of some new sources of dust, which contributed to a substantial increase in dust activity in western Iran in recent years. Our analysis indicates that about 1133.5 km^2^ of regions in the Middle East with permanent surface water in 1984 completely dried up in 2015 and acted as potential sources of dust, while about 2154.2 km^2^ of water bodies dried up in some seasons in 2015 compared to 1984. The highest decrease of water bodies among the main sources of dust belongs to Lake Razzaza (around 50%). Lake Habbaniyah and Lake Tharthar reached their lowest surface water area in 2011, while the lowest surface water area in Lake Hemrin occurred in 2009, and that of Hour al-Azim occurred in 2010 and 2011. The eastern part of Lake Hemrin, the northwestern part of Lake Habbaniyah, the northeastern part of Lake Razzaza, the Shore of Lake Tharthar especially in its northwestern part, the West Hammar Marshes, and the northern part of Hour al-Azim have also dried up from 2008 to 2012. In addition, 726.1 km^2^ of the sources of dust lost their shrub cover and turned into barren lands between 2010 and 2012. About 248.2 km^2^ of permanent wetlands became croplands from 2009 to 2010, while around 80 km^2^ were converted from croplands and grasslands into shrublands from 2010 to 2012. We argue that the emergence of new sources of dust in the Middle East in recent years has largely contributed to an increase in the frequency of dust events in western Iran.

### Supplementary Information

Supplementary Figures.

## Data Availability

Iran’s hourly visibility and the present weather data of the synoptic stations was taken from Iranian Meteorological Organization. MOD04 L2 product of MODIS was taken from https://worldview.earthdata.nasa.gov/. The Hybrid-Single Particle Lagrangian Integrated Trajectory (HYSPLIT) model was taken from https://www.ready.noaa.gov/HYSPLIT.php. The reanalysis Interim (ERA-Interim) data used in this study were obtained from the ECMWF data server on single levels at https://apps.ecmwf.int/datasets/data/interim-full-daily/levtype=sfc/ and pressure levels at https://apps.ecmwf.int/datasets/data/interim-full-daily/levtype=pl/. MODIS land cover product (MCD12C1) was taken from https://modis.gsfc.nasa.gov/data/dataprod/mod12.php. MODIS MOD13Q1 product was obtained from https://developers.google.com/earth-engine/datasets/catalog/MODIS_061_MOD13Q1. MODIS MOD11A2 product was obtained from https://developers.google.com/earth-engine/datasets/catalog/MODIS_061_MOD11A2. Landsat 7 satellite images was obtained from https://developers.google.com/earth-engine/datasets/catalog/LANDSAT_LE07_C01_T1_SR. Precipitation data (TRMM) were taken from https://developers.google.com/earth-engine/datasets/catalog/TRMM_3B43V7.
