# Supplementary material for: Dust in Western Iran: the emergence of new sources in response to shrinking water bodies
Source: Sci Rep. 2023 Sep 27;13:16158. doi: 10.1038/s41598-023-42173-3 (PMC10533835; doi:10.1038/s41598-023-42173-3)
Supplement: Supplementary file 1 — Supplementary Figures. [file 41598_2023_42173_MOESM1_ESM.docx]

**Supplementary Material**

**
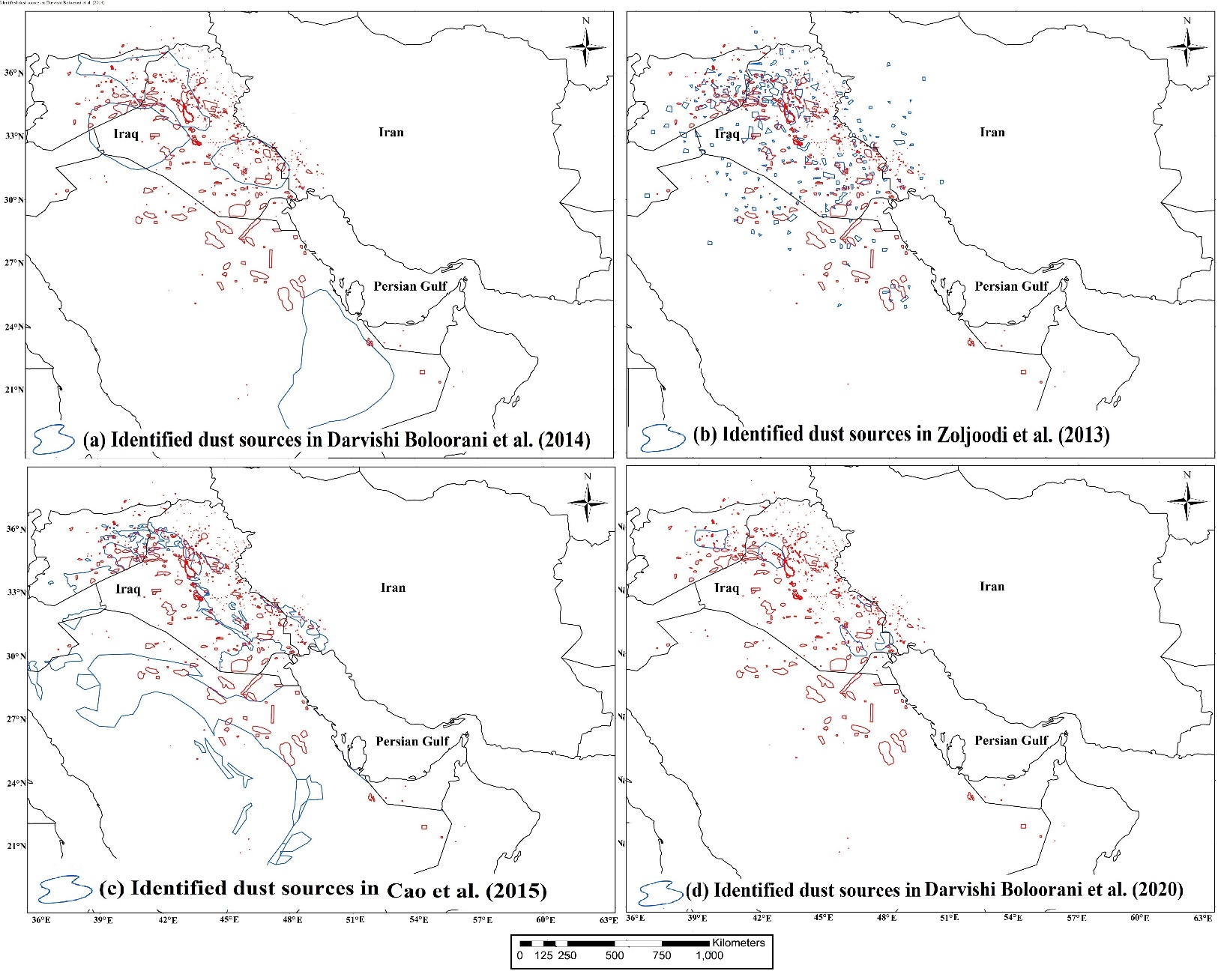
**

Supl. Figure 1. A comparison of dust sources identified in this study (red) and in some other studies (blue), including (a) Darvishi Bollorani et al. (2014), (b) Zoljoodi et al. (2013), (c) Cao et al. (2015), and (d) Darvishi Boloorani et al. (2020).

**
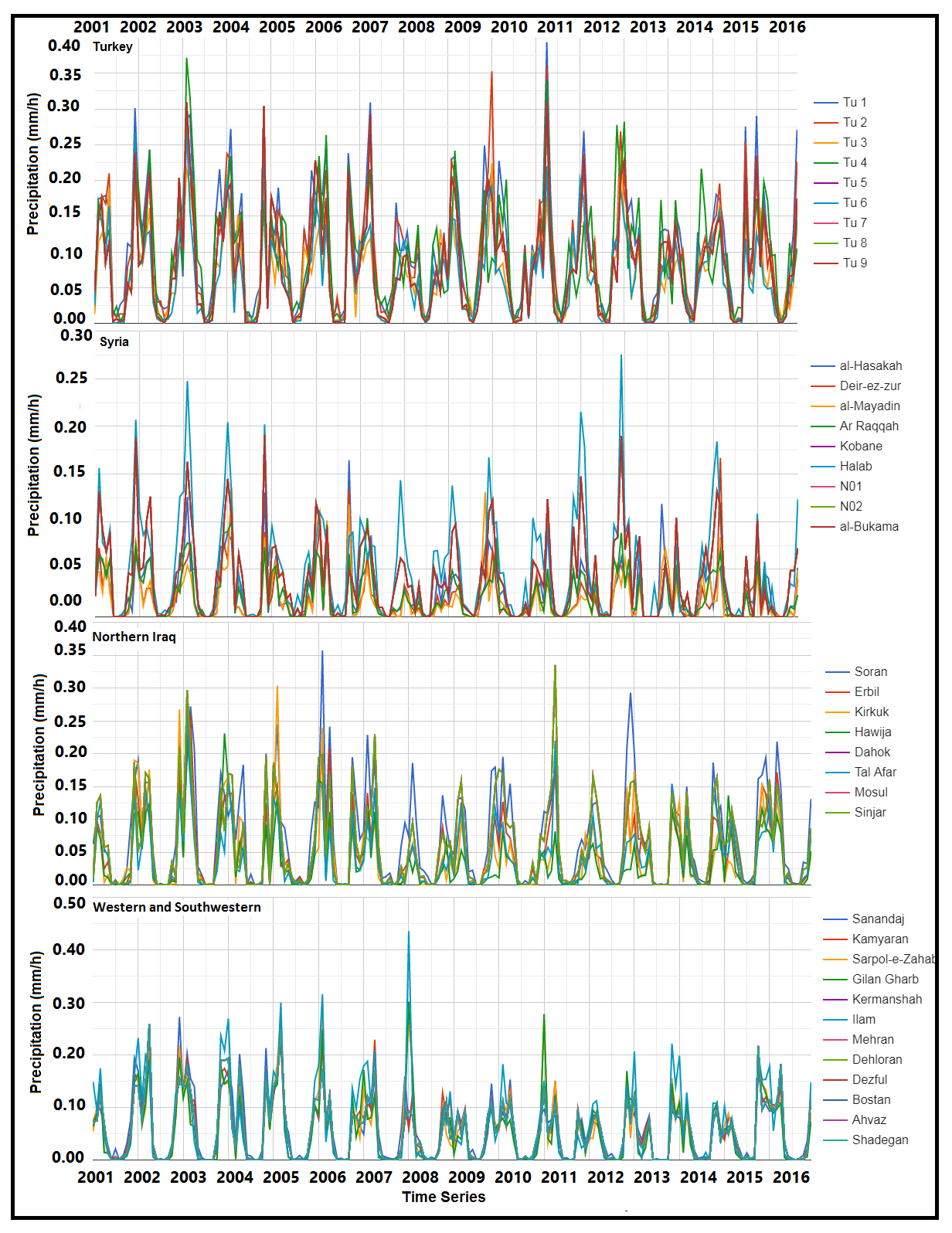
**

Supl. Figure 2. Time series of precipitation in different locations and countries in West Asia. These locations are shown in Figure 1. Precipitation data were taken from the 3B43 algorithm of the Tropical Rainfall Measuring Mission (TRMM) satellite.

**
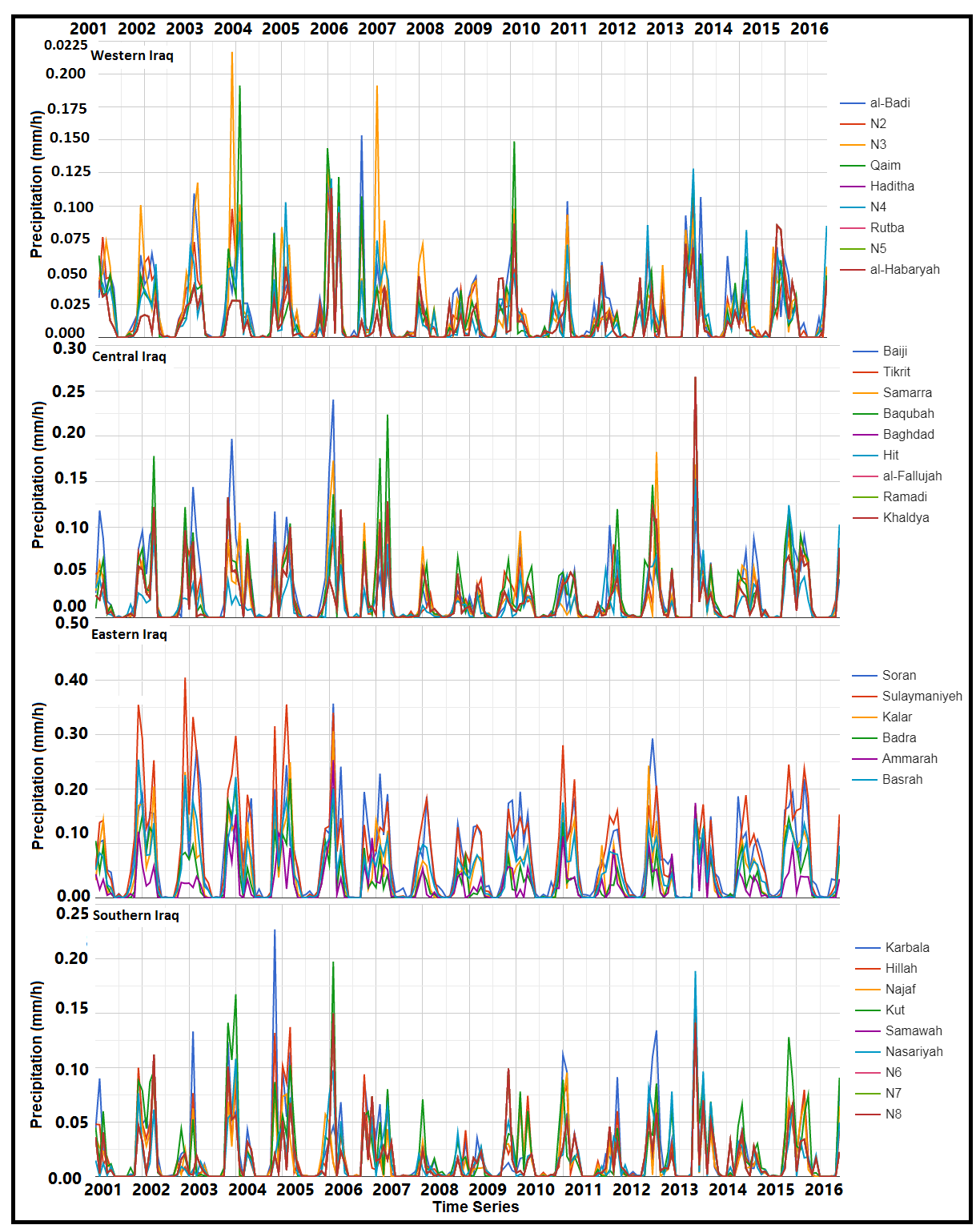
**

Supl. Figure 3**.** Same as Figure A, but for different locations and countries in West Asia. These locations are shown in Figure 1.
